# Whole genome sequencing and microsatellite analysis of the *Plasmodium falciparum* E5 NF54 strain show that the *var*, *rifin* and *stevor* gene families follow Mendelian inheritance

**DOI:** 10.1186/s12936-018-2503-2

**Published:** 2018-10-22

**Authors:** Ellen Bruske, Thomas D. Otto, Matthias Frank

**Affiliations:** 1 Institute of Tropical Medicine, University of Tuebingen, Wilhelmstr. 27, 72074 Tuebingen, Germany; 2 Malaria Programme, Wellcome Trust Sanger Institute, Hinxton, CB10 1SA UK; 3 Present Address: Centre of Immunobiology, Institute of Infection, Immunity & Inflammation, College of Medical, Veterinary and Life Sciences, University of Glasgow, Glasgow, UK

**Keywords:** *var* genes, Recombination, E5, 3D7, NF54, Variant surface antigens, Antigenic variation, Epigenetic, PfEMP1, RIFIN, STEVOR, Microsatellites, Whole genome sequencing, Cross over recombination, Non cross over recombination

## Abstract

**Background:**

*Plasmodium falciparum* exhibits a high degree of inter-isolate genetic diversity in its variant surface antigen (VSA) families: *P. falciparum* erythrocyte membrane protein 1, repetitive interspersed family (RIFIN) and subtelomeric variable open reading frame (STEVOR). The role of recombination for the generation of this diversity is a subject of ongoing research. Here the genome of E5, a sibling of the 3D7 genome strain is presented. Short and long read whole genome sequencing (WGS) techniques (Ilumina, Pacific Bioscience) and a set of 84 microsatellites (MS) were employed to characterize the 3D7 and non-3D7 parts of the E5 genome. This is the first time that VSA genes in sibling parasites were analysed with long read sequencing technology.

**Results:**

Of the 5733 E5 genes only 278 genes, mostly *var* and *rifin/stevor* genes, had no orthologues in the 3D7 genome. WGS and MS analysis revealed that chromosomal crossovers occurred at a rate of 0–3 per chromosome. *var, stevor* and *rifin* genes were inherited within the respective non-3D7 or 3D7 chromosomal context. 54 of the 84 MS PCR fragments correctly identified the respective MS as 3D7- or non-3D7 and this correlated with *var* and *rifin/stevor* gene inheritance in the adjacent chromosomal regions. E5 had 61 *var* and 189 *rifin/stevor* genes. One large non-chromosomal recombination event resulted in a new *var* gene on chromosome 14. The remainder of the E5 3D7-type subtelomeric and central regions were identical to 3D7.

**Conclusions:**

The data show that the *rifin/stevor* and *var* gene families represent the most diverse compartments of the *P. falciparum* genome but that the majority of *var* genes are inherited without alterations within their respective parental chromosomal context. Furthermore, MS genotyping with 54 MS can successfully distinguish between two sibling progeny of a natural *P. falciparum* cross and thus can be used to investigate identity by descent in field isolates.

**Electronic supplementary material:**

The online version of this article (10.1186/s12936-018-2503-2) contains supplementary material, which is available to authorized users.

## Background

The malaria parasite *Plasmodium falciparum* is the most prevalent malaria species found on the African continent [1] and is responsible for 90% of deaths from malaria [2]. The NF54 isolate derives from an infection obtained near Schiphol Airport in the Netherlands [3]. Two sibling parasites, 3D7 and E5, were independently isolated by limiting dilution from the original NF54 culture [3, 4]. The 3D7 clone has been used in the malaria genome sequencing project [5], revealing that the *P. falciparum* genome consists of a 23 Mb nuclear genome with 14 chromosomes and around 5500 genes [6]. E5 was incidentally indentified during transfection experiments of the original NF54 culture [4] and has previously been characterized by PCR cloning and gene specific PCR [7]. Whole genome sequencing of *P. falciparum* is complicated by the special properties of the *P. falciparum* genome: it is very AT-rich and contains many repetitive regions and homopolymer runs, especially in its intergenic regions, complicating the assembly of genome data [8–10]. It has recently been shown that the genome can be divided into a core genome (95%) hypervariable regions (5%) [10, 11]. Unambiguous alignments of sequence data of different strains are only possible in the core regions and not in the hypervariable regions that harbour the majority of the variant surface antigen (VSA) gene families [12–14].

To date, five multicopy gene families that encode VSAs have been described in *P. falciparum*: *stevor* (subtelomeric variable open reading frame) [15], *rif* (repetitive interspersed family) [16], *pfmc*-*2tm* (*P. falciparum* Maurer’s clefts two transmembrane) [17], *surfin* (surface associated interspersed genes) [18] and *var* [19]. The best investigated VSA is *P. falciparum* erythrocyte membrane protein 1 (PfEMP1) [6, 20, 21]. PfEMP1 is encoded by the multicopy *var* gene family that consists of about 60 *var* (variability) genes per *P. falciparum* genome [19]. Antigenic variation is primarily mediated by mutually exclusive expression of 1 of the 60 *var* genes per infected red blood cell. The subtelomeric position of most *var* genes [14] predisposes them to recombination contributing to the diversity of PfEMP1 [22]. PfEMP1 is transported to the surface of infected red blood cells and acts as a receptor for the surface receptors on endothelial host cells. This cytoadhesion prevents clearance of the red blood cells by the spleen. Different forms of PfEMP1 possess different binding specificities and individual PfEMP1 variants have been associated with distinct malaria syndromes such as malaria in pregnancy or cerebral malaria [23–28].

In endemic regions, antibodies to PfEMP1 develop early in life have and have been shown to correlate with the development of protective immunity [29]. To escape the human immune response, *P. falciparum* can switch the PfEMP1-variant expressed on the surface of infected red blood cells. Recent investigations also support a role for the non-PfEMP1 VSA proteins in cytoadhesion, antigenic variation and as targets of the human immune response [30–32]. The non-PfEMP1 VSA families are located in close proximity to the *var* genes within the hypervariable regions of the *P. falciparum* chromosomes. The chromosomal position of the VSA gene families thus complicates their genetic analysis. Because of this position the VSA gene families were excluded from a recent extensive analysis of progenies of experimental *P. falciparum* crosses [10].

The aim of this work was to characterize VSA-gene family inheritance in a NF54 clone with WGS technology. To provide a framework to investigate identity by descent (IBD) in field isolates a set of 84 microsatellites was evaluated for its ability to distinguish between the 3D7 and non-3D7 parts of the E5 genome. Microsatellites are variable numbers of tandem repeats in DNA [33]. They have the advantage that they are locus-specific and highly polymorphic. Because most microsatellites are located in non-coding regions they are not subject to purifying selection. The original work by Walliker, Wellems and Su has generated a large repository of MS primers that were originally used to determine the genetic basis of chloroquine resistance [34] and erythrocyte invasion in progeny of experimental genetic crosses [35] as well as multiple other fundamental aspects of *P. falciparum* biology (summarized in Figan et al. [36]). MS flanking drug resistance loci have also been employed to determine the size of genetic sweeps in population based studies [37, 38]. A 12-locus primer set developed by Anderson et al. [39] has been used by many investigators to assess the genetic diversity of field isolates [40, 41]. Recently, Figan et al. [36] identified 12 MS markers that can reliably differentiate progeny from experimental crosses. However, the small number of MS precludes an analysis of chromosomal inheritance. Therefore, here we evaluate a set of 84 microsatellite alleles distributed over the 14 *P. falciparum* chromosomes to type chromosomal regions as 3D7- or non-3D7.

Genome changes in progeny of a *P. falciparum* cross are a consequence of crossover or non-crossover recombination [10, 42]. Crossover recombination represent, a reciprocal exchange between homologous chromosomes during meiosis, whereas non-cross over recombination results in the duplication of a sequence from a donor sites that replaces a sequence at an acceptor site (also referred to as a gene conversion).

The analysis of E5 offered the opportunity to investigate crossover and non-crossover recombination in a natural sibling of the 3D7 genome clone. Zero to three cross- overs per chromosome were identified. VSA gene families were inherited in their respective parental chromosomal background. The chromosomal distribution of VSA genes in E5 was virtually identical to 3D7. The *var* and *rifin/stevor* gene families represented the most genetically distinct parts of the E5 genome. However, only one definite non-crossover recombination event among non-3D7 and 3D7 *var* genes was detected.

## Methods

### Parasites, cell culture and generation of DNA

The NF54-C2 clone (isogenic with 3D7) [43, 44] and the NF 54 E5 [4] clone were used for in vivo microsatellite typing. Parasites were cultivated in RPMI 1640 medium completed with 10% Albumax concentrate (Gibco), 2 mM Glutamine, 0.05 mg/ml Gentamicin and 25 mM Hepes buffer (Sigma) at 2.5% haematocrit of 0+ erythrocytes from a local blood bank. Culture flasks were kept at 37 °C under standard parasite cell culture conditions (5% O_2_, 5% CO_2_, 90% N_2_). At a parasitaemia of ca. 4%, the erythrocytes were spun down and parasite DNA was extracted from the pellet using the QiAmp DNA Blood Midi Kit according to the manufacturer’s manual.

### *var* gene PCR and Sanger sequencing

PCR using *var*-specific primers from Salanti et al. [26] with modifications [44] was carried out using the following conditions: 94 °C 3 min, 94 °C 30 s, 48 °C 45 s for 30× cycles, then 70 °C 30 s, 70 °C 3 min. PCR products were run on a 1% agarose gel, bands were cut out and purified with NucleoSpin^®^ Extract Kit (Macherey–Nagel) according to the manufacturer’s protocol. Preparation of the DNA for sequencing was as follows: the reaction mix containing 1× sequencing buffer, 10% Big Dye Terminator v1.1 (Applied Biosystems), sequencing buffer (Applied Biosystems), 125 µM primer, 60% dH_2_O, 1–5 ng DNA were run with the following conditions: 94 °C 10 s, 50 °C 5 s, 25 cycles, 60 °C 4 min. All samples were purified using a 6.7% Sephadex (w/v) column. Sequences were aligned and a consensus sequence was generated with the help of BioEdit sequence alignment editor (http://www.mbio.ncsu.edu/BioEdit/bioedit.html).

### Microsatellite primer selection

For each of the 14 chromosomes microsatellites (MS) were selected with the help of the NCBI map viewer database (http://www.ncbi.nlm.nih.gov/mapview/maps.cgi). The initial selection of MS was based on the unpublished analysis of the 3D7xHB3 cross (kindly provided by Akhil Vaidya, Department of Microbiology and Immunology, Drexel University College of Medicine, Philadelphia, Pennsylvania, USA). A total of 84 MS were evaluated. In order to allow cross reference to MS alleles employed in other MS genotyping investigations 4 microsatellites evaluated by Anderson et al. [39] were also included. Subsequently, 4 MS per chromosome were selected for the genotyping of E5 and NF54C2. In general, 3 MS were located in subtelomeric regions and 1 in a central chromosomal region. For chromosome 7, 5 MS were chosen. The MS 9B12, located 1.4 Kb downstream the chloroquine (CQ) resistance gene *pfcrt* [38] and MS B5M77, which is located 18.1 Kb upstream of the *pfcrt* locus were also included. 9B12 is highly conserved in chloroquine resistant strains. B5M77 is positioned at the 3′ end of the “chloroquine resistance genetic sweep” area and thus exhibits low allele diversity in resistant strains but high allele diversity even in chloroquine sensitive strains [38, 45].

### Microsatellite PCR

Microsatellite PCR was performed using the following conditions: 5 µl DNA were used in a 50 µl reaction containing 1× PCR buffer, 1.5 mM MgCl_2_, 0.08 µM dNTPs and 0.25 µM primer. For fragment analysis, the forward primer was labelled at its 5′ end (Eurofins Genomics). Four different dyes were used per chromosome (Table 1). The program was as follows: 94 °C 5 min, 94 °C 20 s, 45 °C 10 s, 40 °C 10 s, 60 °C 30 s, 40×, 65 °C 2 min. PCR products were checked on a 1.5–2% agarose gel.

**Table 1:**
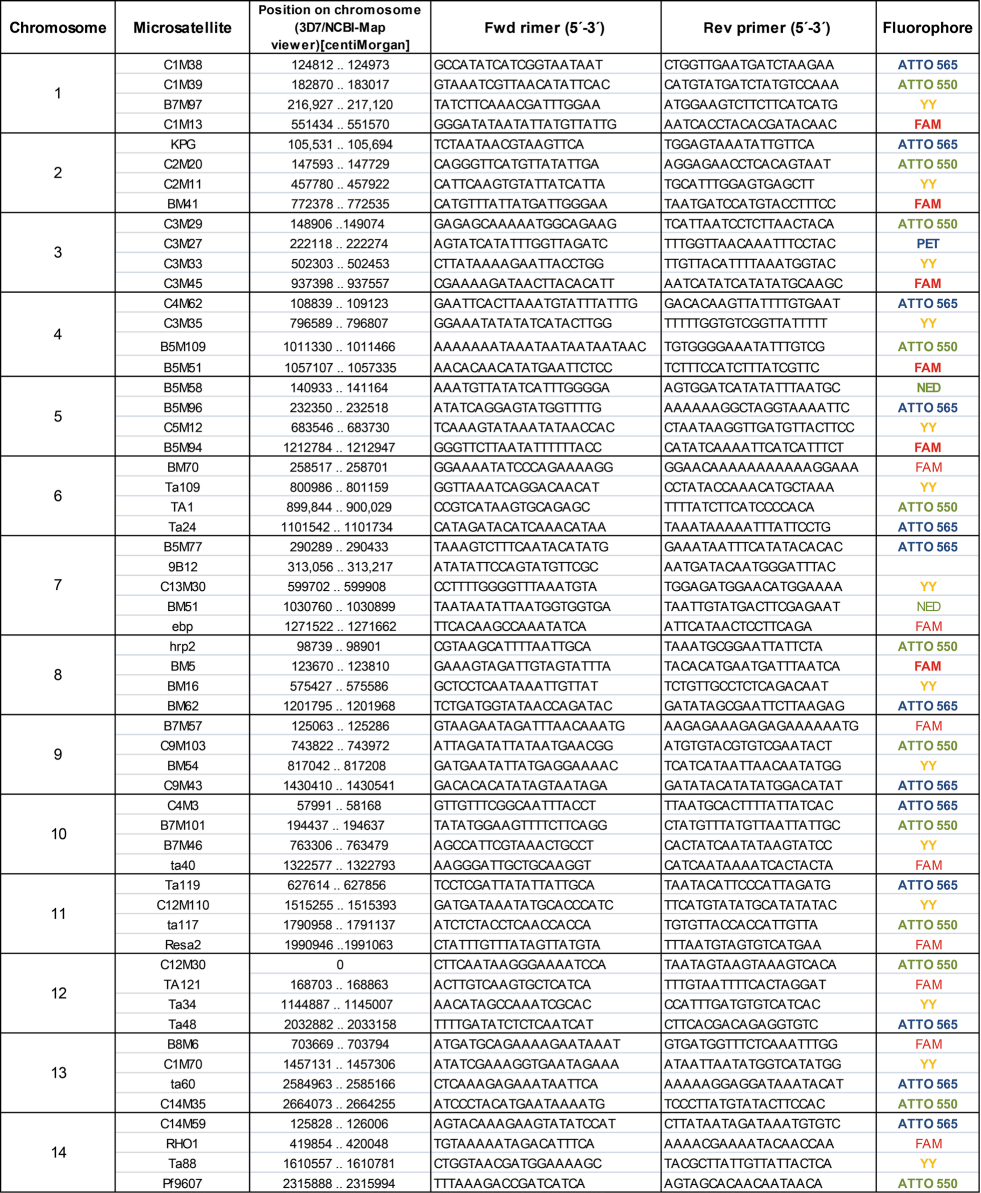
Microsatellite coordinates on the respective chromosomes, primer sequences and the dyes used are depicted

### MS sequence analysis

To verify the MS position, all MS were amplified from NF54 C2 DNA and the reaction products were sequenced. The obtained sequences were manually aligned with the 3D7 MS sequence in the database. Only MS PCR primers pairs that amplified the 3D7 reference sequence were used for the fragment analysis.

### Fragment analysis

PCR products were all diluted 1:200 with water. A master mix was prepared with 5 µl H_2_O and 5 µl Formamide (Hi-Di Life Technologies) and − 0.1 µl Standard (LIZ 500 Life Technologies). Per singleplex reaction 10 µl master mix and 1 µl diluted PCR product were used. For multiplex fragment analysis 10 µl master mix and 1 µl diluted PCR product of each PCR reaction were used. The mix was heated at 95 °C for 3 min and immediately chilled on ice for a few minutes before fragment analysis. Analysis was done in 96-well plates (Biozym Scientific GmbH) with the Applied Biosystems ABI Prism 3130xl Genetic Analyzer and data were evaluated using GeneMapper v 4.1.

### Chimera breakpoint PCR

PCR across the breakpoint of the chimeric *var* gene in E5 containing an E5-like half downstream and a part (approx. 3 kb) of the 3D7 *var* gene Pf3D7_083350 upstream was done using the primers PFE5_F1 (5′-CGCCATAGTATCACCAATGC-3′) and PF3D7083350_R2 (5′-CCCGACGTGGTACACCTG-3′) with the following conditions: 3 min 94 °C, 10 s 94 °C, 30 s 56 °C, 30 s 72 °C, 3 min 72 °C, 40 cycles, using 5 µl DNA template (concentration up to 5 ng), 2.5 µM primer, 0.2 mM MgCl_2_, 0.2 mM dNTPs. PCR products were checked on an 1% agarose gel.

### Whole genome sequencing

#### Illumina

Genomic DNA of E5 was sheared into 250–350 bp fragments by focused ultrasonication [Covaris Adaptive Focused Acoustics technology (AFA Inc., Woburn, USA)]. An amplification-free Illumina library [46] was prepared and sequenced on a Illumina GAII (150 bp) platform according to the manufacturer’s standard sequencing protocol. Reads were mapped with smalt (ftp://ftp.sanger.ac.uk/pub/resources/software/smalt/,parameter–x–a 1000). Variants were called with gatk against the *P. falciparum* version 3 assembly from geneDB [47, 48].

#### Pacific bioscience reads

From the same DNA, SMRTbell template library using the Pacific Biosciences issued protocol (20 kb Template Preparation Using BluePippin Size-Selection System) were generated. Five SMRT cells were sequenced on the PacBio RS II platform using P5 polymerase and chemistry version 3. Raw sequence data were deposited in the European Nucleotide Archive under accession number ERS500965.

#### Sequence processing

Sequence data from the SMRT cells were assembled with HGAP. As expected genome size 23.5 Mb was used. Next, the contigs were further improved with IPA (https://github.com/ThomasDOtto/IPA). The script performs following steps: delete small contigs, identify overlapping contigs with low Illumina coverage, order contigs against the *P. falciparum* 3D7 reference using ABACAS2 [49], corrects errors with Illumina reads using iCORN2 [50], circularizes the two plastid genomes with circulator [51] and renames the chromosomes and contigs. The draft genome was annotated with companion [52], using *P. falciparum* 3D7 version 3 from October 2015 as reference.

#### Bioinformatic sequence analysis

Using Artemis [53] and bamview [54], a free genome browser and annotation tool, and the Artemis Comparison Tool (ACT) [54, 55], a pairwise comparison tool of DNA sequences from the Wellcome Trust Sanger Institute (Hinxton, UK) to visualize similarities and differences between genomes, the genome of 3D7 (serving as reference) was compared with E5. By laying one sequence over the other, coverage and single nucleotide polymorphism (SNP) maps of the E5 reads over the 3D7 genome can be loaded into the program. The SNP map served as a tool to detect differences between 3D7 and E5. Areas with low SNP frequency and even Illumina read coverage were defined as 3D7 chromosomal areas. To find shared proteins between PfE5 and Pf3D7, they were compared (ignoring alternative splicing) with a BLASTp (E-value cutoff 1e−6) and then clustered with orthomcl version 1.4, default parameter [56].

## Results

### Analysis of chromosomal inheritance in E5

To characterize chromosomal regions of E5 as 3D7 or non-3D7 a set 84 MS primer pairs was evaluated for multiplex PCR with fluorescent probes. First, all MS were amplified under the same set of PCR conditions from 3D7 DNA. Most of the reactions resulted in a clear product after gel electrophoresis. Sanger sequencing and manual alignment of the sequences to the 3D7 reference genome showed that 57 of the 84 MS PCR fragments could be unambiguously aligned to the target MS sequence, whereas 27 MS resulted in sequences that either did not amplify or could not be aligned with the target sequence (Additional file 1).

57 MS were, therefore, employed for genotyping (Table 1). All MS were amplified from E5 and 3D7 DNA and MS length was determined by capillary fragment analysis. Each chromosome was characterized at 4–5 MS loci. An MS allele was designated as 3D7-type, if the fragment length difference was ≤ 3 bp between E5 and 3D7. E5 had 37 3D7-type and 20 non-3D7 MS alleles (Table 2). On chromosomes 1, 3, 6 and 9 all MS alleles were 3D7-type. The remaining 10 E5 chromosomes were composed of 3D7 and non-3D7 MS alleles. The pattern of MS allele distribution suggested that large chromosomal haplotypes were inherited together, however the distant spacing of the MS markers precluded fine scale mapping. The latter was only achieved for the area of chromosome 7 that harbours the chloroquine resistance gene *pfcrt*. The microsatellites 9B12, located 1.4 Kb downstream of *pfcrt* [34, 45] and B5M77, located 18.1 Kb upstream of this locus, had 3D7-type alleles in E5. Thus, MS in the “genetic sweep” area were the same in E5 and 3D7, suggesting that in both clones this chromosomal fragment was inherited as a 3D7-type haplotype.

**Table 2:**
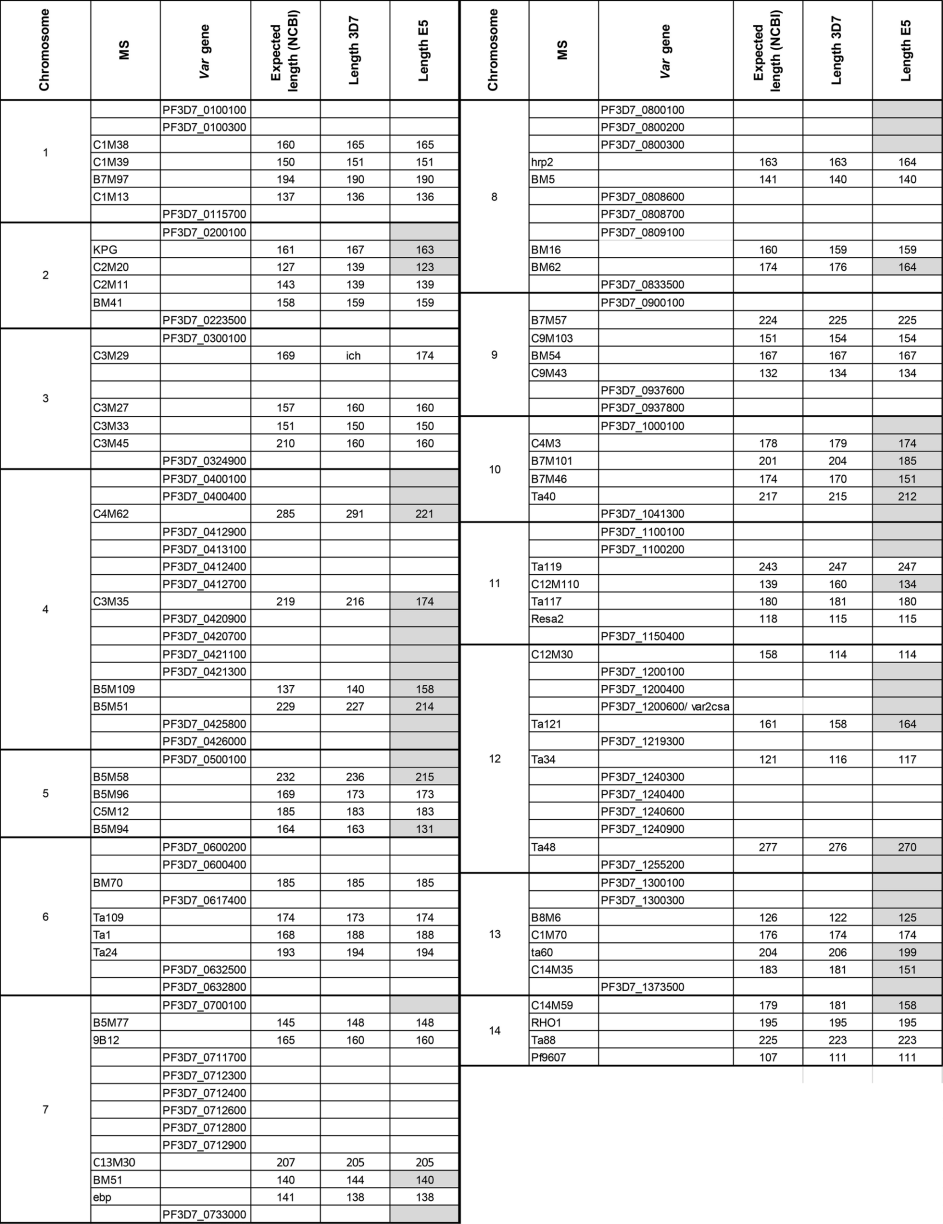
Genotyping based on microsatellite fragment length and 3D7 specific *var* gene PCR

The MS individual fragment lengths are shown for 3D7 and E5 as well as for the in silico 3D7 microsatellite lengths in the NCBI database. MS alleles with > 3 bp size difference between 3D7 and E5 are typed as non-3D7 (grey). 3D7 alleles are white, non-3D7 alleles are grey. 3D7 *var* gene amplification on E5 DNA was verified by targeted Sanger sequencing of PCR fragments. Note that the table differs from Table 1 in Frank et al. [7] at the 5′ end of Chromosome 7 (reannotation of PF3D7_0700100 previously Mal8P1.220) and at the 3′ end of Chromosome 13: PF3D7_1373500 (previously MAL13P1.356)

To further evaluate chromosomal inheritance we next characterized E5 and 3D7 with a 3D7 specific *var* gene primer set. This revealed overall good correlation between the presence of 3D7 *var* genes and 3D7 MS alleles in the respective chromosomal areas (Table 2). However, in a few telomeric areas MS and *var* gene typing did not correlate. This was most noticeable on chromosome 7 (*ebp*), 8 (*hrpII*) and 12 (C12M30) where the MS genotyping suggested a 3D7 chromosomal haplotype but the adjacent 3D7 *var* genes were not amplified from E5.

Comparison of the PCR fragment lengths of the 57 MS after amplification on 3D7 DNA with the “in silico” length of the respective MS in the 3D7 genome (version 3) revealed, that 21 MS exhibited a size difference of > 3 bp thus raising the question of the validity of some of the MS typing results.

**Fig. 1:**
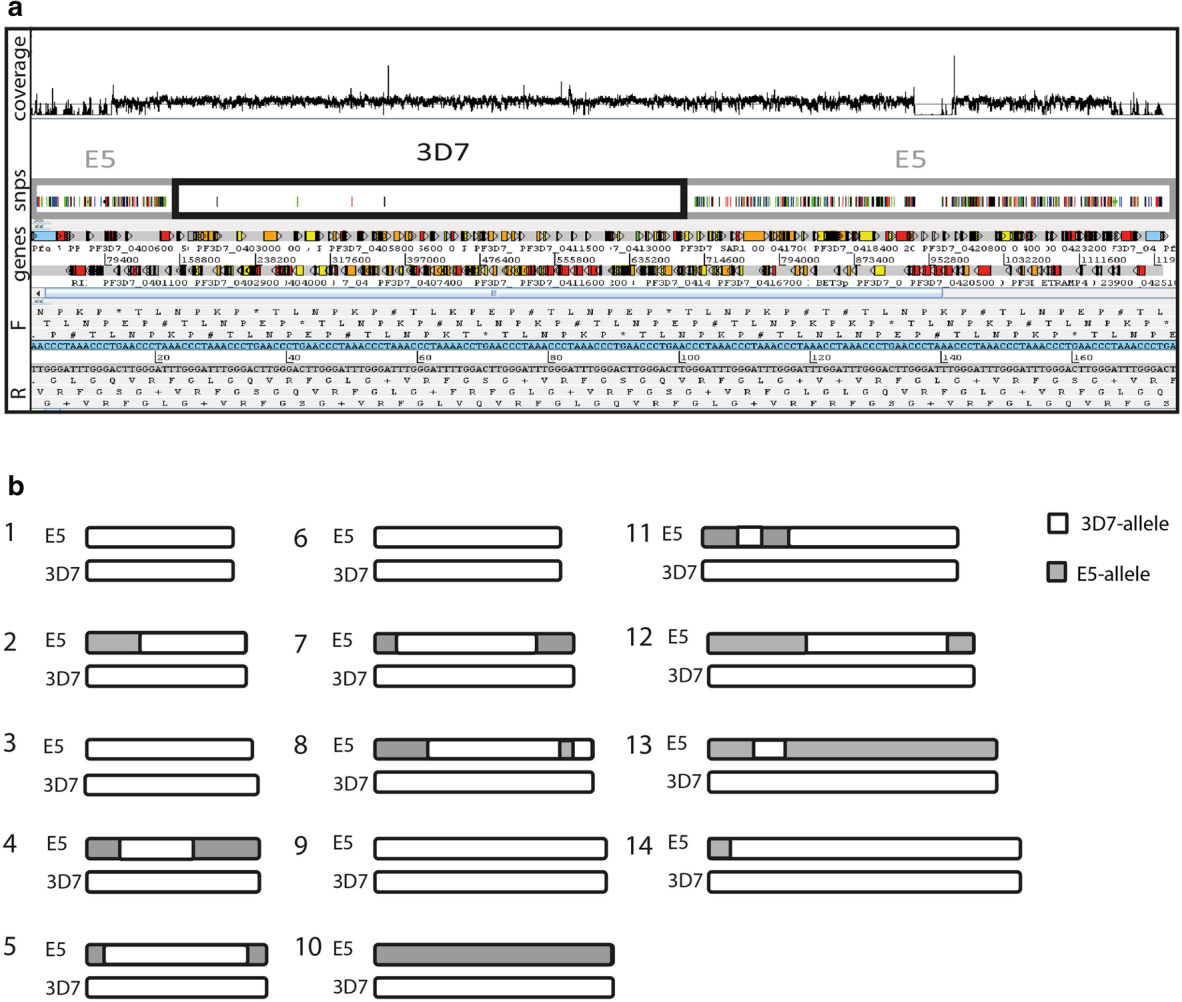
**a** 3D7 Artemis view of chromosome 4 showing snp plots and coverage in comparison to E5. Areas with many SNPs and low coverage were defined as non-3D7 (E5-type), those with only few SNPs and a high coverage were regarded as 3D7-type. **b** Chromosome map deriving from illumina whole genome sequencing data showing putative crossovers in the individual chromosomes of 3D7 compared to E5. 3D7-alleles are depicted in white, parts distinct from 3D7 (“E5-alleles”) in grey

To validate the MS and *var* gene haplotyping results E5 was characterized by whole genome Illumina sequencing resulting in a E5 genome with > 100× coverage. Mapping of E5 onto the 3D7 reference sequence revealed areas with many SNPs and low coverage, which were defined as non-3D7 (E5-type) and areas with only few SNPs and even median coverage which were regarded as 3D7-type (Fig. 1a). Fragment analysis as well as WGS both showed the same pattern of cross overs in chromosomes 1–14 in clone E5 (compared to 3D7 reference) (Fig. 1b) and confirmed that chromosomes 1, 3, 6 and 9 of E5 were identical with 3D7 and thus appeared to have been inherited without cross overs. In the remaining 10 chromosomes between one to three cross overs per chromosome were detected by Illumina WGS. WGS haplotyping of the telomeric areas of chromosome 7, 8 and 12 typed these areas as non-3D7, confirming the *var* PCR genotype. In contrast MS genotyping of the same areas (*ebp*, *hrp2* and C12M30) showed identical alleles between E5 and 3D7 suggesting a 3D7-haplotype. Together the data suggested that these 3 MS were not sufficiently diverse to allow haplotyping of sibling parasites and they were, therefore, excluded from further analysis. In summary, 54 MS allele typing results were confirmed by WGS as being 3D7 or non-3D7. Overall, the MS and WGS data showed that genetic exchange in the progeny of a natural genetic cross occurred at a rate of 0–3 crossover per chromosome. Allthough *var* gene fragment haplotyping correlated well with microsatellite and WGS haplotyping the Illumina read length precluded a exact anaylsis of VSA gene family inheritance in E5.

### Anaylsis of VSA gene family inheritance in E5

Short read assemblies do not permit the accurate assembly of non-reference subtelomeric regions, therefore VSA family inheritance in E5 was assessed by long read Pacific Bioscience sequencing technology. This resulted in an assembly of 58 contigs. Using IPA, the number of contigs was reduced to 29, including the apicoplast and mitochondrial genomes, resulting in a total assembly length of 23.3 Mb.

**Fig. 2:**
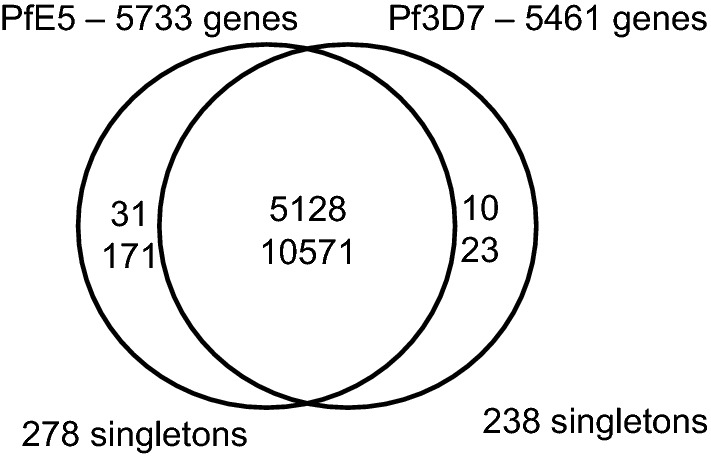
Venn diagrams displaying shared and species-specific orthologue clusters and their proteins in the target genome *P. falciparum* E5 and the *P falciparum* 3D7 reference. Singletons, i.e. genes without orthologues and paralogues in either species, are placed outside the Venn diagram to the left and right. The numbers within the Venn diagram that belongs to both genomes represent the number of orthologue groups (upper number) and the number of genes in orthologue groups (lower number). The numbers within the E5 and 3D7 specific Venn diagram circles represent paralogue groups and the number of genes within paralogue groups. The assembly can be found at ftp://ftp.sanger.ac.uk/pub/project/pathogens/Plasmodium/falciparum/E5/Version1

Annotation of the assembly generated 5733 genes (Table 3). Overall the E5 genome showed a highly conserved structure compared to the 3D7 reference genome. Of the 5733 E5 genes all except 278 had orthologues in the 3D7 genome. These 278 genes were, therefore, designated as singletons (Fig. 2). The *rifin/stevor* and *var* families had 58 singletons and 11 singletons respectively and together represented the largest group of genes with known function among the singletons (Additional file 2).

**Table 3:** *Plasmodium falciparum* NF54 E5 genome characteristics

Number of annotated regions/sequences	29
Number of genes	5733
Gene density (genes/megabase)	240.97
Number of coding genes	5607
Number of pseudogenes	126
tRNA	105
Overall GC%	19.28
Coding GC%	23.9

**Fig. 3:**
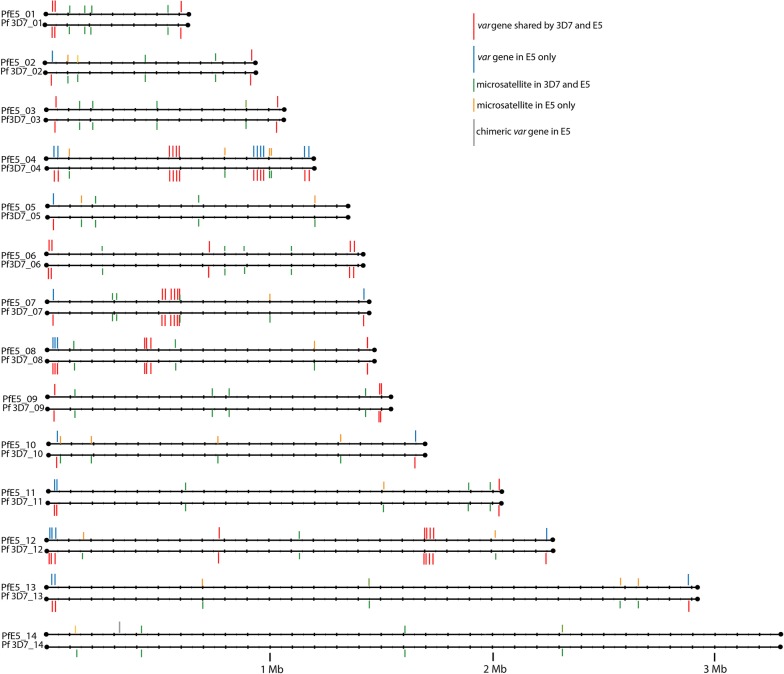
E5 and 3D7 have the same *var* gene distribution into telomeric repeats and central clusters. *var* genes that are identical between E5 and 3D7 are coloured in red. *var* genes only found in E5 are blue. MS that are identical between E5 and 3D7 are coloured in green. MS that are only found in E5 are coloured in orange. The E5 chimeric *var* gene on chromosome 14 is depicted in grey. Note that for clarity reasons only the *var* genes are depicted. The exact chromosomal location of the *rifin/stevor* gene positions can be found at ftp://ftp.sanger.ac.uk/pub/project/pathogens/Plasmodium/falciparum/E5/Version1

The Pacific Bioscience genome assembly confirmed the Illumina haplotyping and furthermore enabled a detailed analysis of the chromosomal areas harbouring VSA gene families. VSA family genes consisted of: 62 full length *var* genes (61 LARSFADIG motifs), 32 *var* pseudo-genes, 189 *rifin*/*stevor* genes, 20 *rifin*/*stevor* pseudo-genes, 8 *surfins*, 6 *Pfmc*-*2TM* genes and 2 *Pfmc*-*2TM* pseudo-genes. Comparison of the Pacific Bioscience E5 genome with the 3D7 genome showed that the VSA antigen families have virtually the same size (Table 4). Both clones share the same distribution of VSA genes into subtelomeric and central regions as 3D7 (genome sequence vs.3) (Fig. 3). One miss-assembly in the first *var* gene cluster of chromosome 4 was detected (Additional file 3).

**Table 4:** 3D7 and E5 VSA-gene families

VSA-gene family	Genes in 3D7	Genes in E5
*var* (≥ *4* kb)	61	62
*rifin *+ *stevors*	190	189
*pfmc*-*2tm*	12	6
*Surfin*	7	8

3D7 data was retrieved from GeneDB [47]

3D7 VSA genes were surrounded by 3D7-type chromosomal areas. Similarly, the non-3D7 (E5 specific) VSA sequences mapped to non-3D7 chromosomal areas. Interestingly for the E5 part that is identical to 3D7 the majority was co-linear with the respective areas in 3D7. Only one large scale recombination event was detected in the 3D7-type subtelomeric regions of E5 (see below).

Based on the Pacific Bioscience assembly the correlation of telomeric MS genotype and *var* gene genotyping was reevaluated (Fig. 3). Of the 54 MS, 27 were located in vicinity of telomeric regions (distance range 50–1,000,000 kb from the telomeres). 11 MS carried 3D7 alleles and 16 non-3D7 alleles. In 26 of the corresponding 27 telomeric areas the *var* gene alleles correlated with the MS alleles. The only exception was the 5′ end of chromosome 11 that carried non-3D7 *var* genes but the next MS TA119 located at approximately 600 kb had a 3D7 allele. WGS showed that a chromosomal crossover had occurred 5′ to TA119. WGS and MS data thus clearly showed that *var* gene inheritance followed a Mendelian pattern.

To estimate the contribution of non-crossover recombination to VSA diversity the *var* gene family of E5 was evaluated. E5 specific *var* genes that shared sequences between 50 and 500 bp with 3D7 were identified and then manually verified using the ACT program. This identified one previously described *var* gene in E5 (PfE5_120005800) that shares 105 bp with Pf3D7_0937800 that is present in E5 and 3D7 on chromosome 9.

**Fig. 4:**
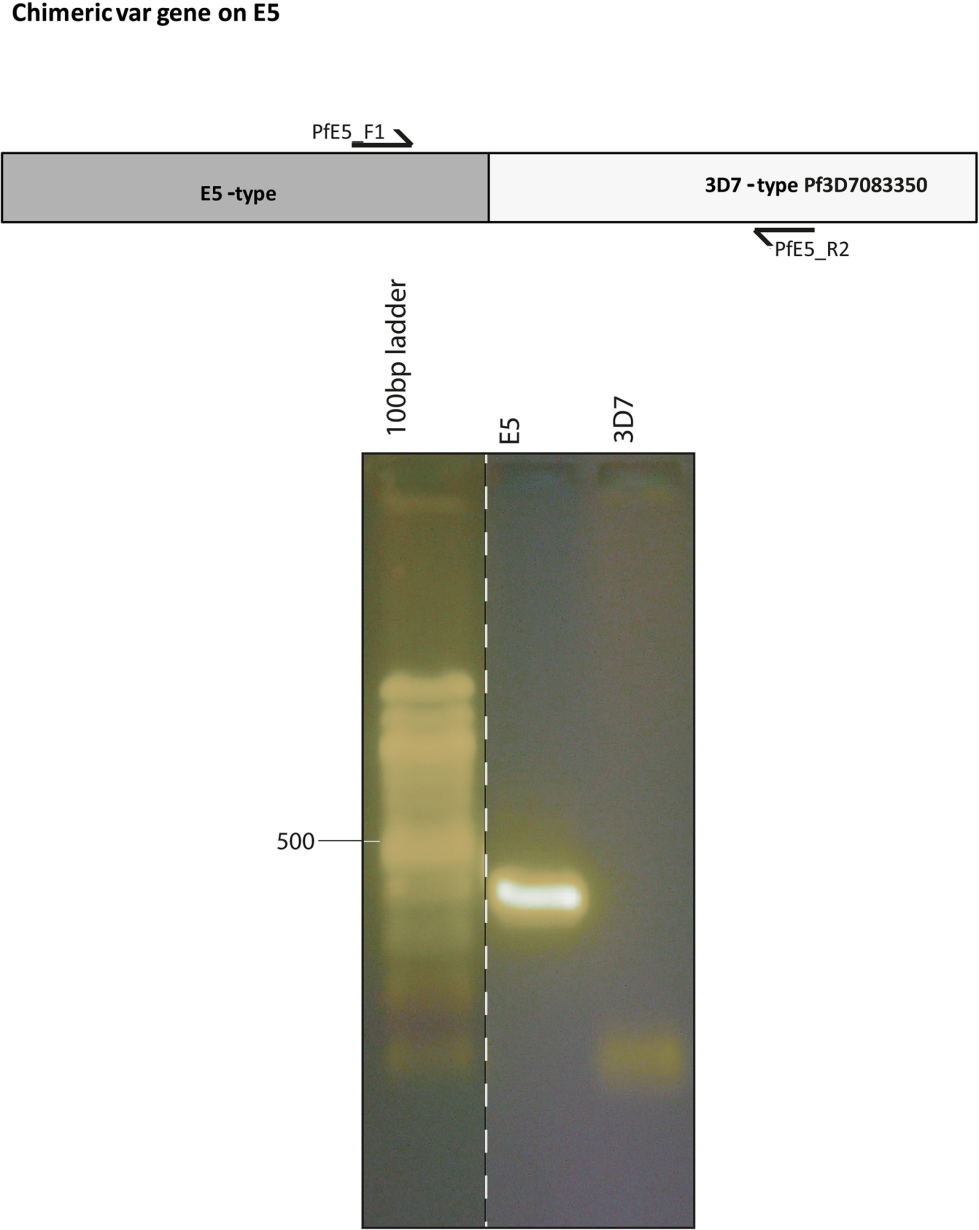
New chimeric *var* gene on chromosome 14 of strain E5. The upper part of the figure displays the localisation of primers F1 and R2 to amplify the crossing point with PCR. The lower part of the figure displays the reaction product after amplification on E5 DNA. No reaction product was obtained after amplification on 3D7 DNA. The order of the gel was rearranged for reasons of clarity and is marked by a dotted line

A new chimera (preliminary nomenclature: PfE5_232200) was located on chromosome 14. It shares one half of exon 1 of the *var* gene Pf3D7_0833500 (MAL7P1.212, approx. 3 kb) and the remainder of the subtelomeric area with 3D7. The rest of the *var* gene is E5-specific (Additional file 4). The same but complete *var* gene Pf3D7_0833500 is also found on chromosome 8 of 3D7 and E5. PCR analysis across the breakpoint in E5, coming from the E5-specific part of the *var* gene and going into the 3D7-type *var* gene, yielded a product and thus verified the result in vivo (Fig. 4). Together the data suggest that the telomeric end of chromosomes 14 up to the middle of the *var* gene is duplicated. Both chimeric genes thus appear to have resulted from a partial duplication of a 3D7 *var* gene.

## Discussion

3D7 and E5 were both cloned from the original NF54 isolate [3, 4] and thus represent progeny of a natural genetic cross. Although the parents of this cross are not known, a previous analysis of 32 progeny of the 7G8XGB4 experimental cross [57] has shown that the two parental genomes are inherited on average at a ratio of 1:1 per progeny. Given that approximately 50% of the E5 genome is identical to 3D7 this suggests that 3D7 is isogenic with one parent of this cross. Thus, analysis of E5 allowed an assessment of chromosomal crossovers as well as non-crossover recombination in a progeny clone of a natural genetic cross.

In this work, the E5 genome was characterized with MS genotyping as well as short and long read WGS techniques. All genotyping approaches suggested a chromosomal recombination rate of 0–3 crossovers per chromosome, consistent with previously reported crossover rates in progeny of experimental genetic crosses [10, 57]. Similarly, all methods indicated that inheritance of VSA gene families occurred within the context of the respective parental haplotypes. A comprehensive analysis of VSA inheritance was however only possible with long read Pacific Biosciene WGS, because the readlength of > 8000 base pairs enabled an accurate assembly of the highly variable telomeric and central chromosomal parts that harbour the VSA gene families. This analysis showed that the VSA gene families have almost the same number of genes in E5 and 3D7.

Annotation of the E5 genome revealed a total of 5733 genes. This number is slightly higher than the 5500 genes in the 3D7 reference genome and is explained by the fact that companion annotation tool overpredicts open reading frames [52]. Genome wide comparison by orthomcl-analysis revealed that the E5 and 3D7 genomes consisted of > 95% genes that had orthologues in both genomes. Only approximately 4% of the E5 and 3D7 genes were singletons and the *rifin/stevor* and *var* genes represented the largest group of genes with known functions among the singletons. Despite this, the total number of identified singleton *var* genes was lower in the orthomcl-analysis than the number of unique E5 *var* genes identified by direct sequence alignment. The underestimation of *var* gene diversity by the orthomcl-analysis is likely due to highly conserved exon II sequences. Overall the data are clearly consistent with the previously reported high genetic diversity of VSA gene families compared to the highly conserved *P. falciparum* core genome.

The *var* gene family has long been shown to be prone to recombination during meiosis [7, 42, 58–60] and mitosis [9, 61, 62]. Furthermore, several investigations have recently quantified mitotic *var* gene recombination rates [9, 62] in different strains. Analysis of the 3D7 and E5 genomes revealed that E5 had a total of 62 *var* genes (compared to 61 *var* genes in the 3D7 reference genome). The “additional” new *var* gene was generated by recombination between a 3D7 *var* gene on chromosome 8 and an E5 specific *var* gene on chromosome 14. 3D7 has no full *var* gene on chromosome 14, but recently Otto et al. showed that 8 of 10 field isolates carry a *var* gene in this subtelomere of chromosome 14 [11]. This shows that non-chromosomal recombination can expand the *var* gene repertoire of individual strains but that the sites of these changes appear to be conserved across different isolates. The presence of an intact “3D7 donor” sequence suggests that the chimeric *var* gene is the result of a gene conversion event as it has been reported previously for the *var* gene family [42, 61]. Recently Calhoun et al. [63] showed that experimentaly induced double stranded breaks are repaired by the “telomerase healing” pathway. Indeed their work showed a similar non-crossover recombination event resulting in the replacement of a chromosome 13 telomere by a chromosome 9 telomere, thereby creating a new chimeric *var* gene on chromosome 13. The data presented here thus support a role for telomere healing in the generation of VSA gene family genetic diversity. A previously described chimeric *var* gene sequence [7] that carries a 105 bp 3D7 fragment within the DBL of the E5 *var* gene was reidentified in the current analysis and the corresponding “3D7 donor” *var* gene was localized to chromosome 9. This chimeric sequence is located within a hypervariable DBL block that has been shown to exhibit high sequence variability in field isolates [64]. Larger population based studies with long read WGS are necessary to determine if this type short chimeric sequence represent true non-chromosomal recombination or simply random sharing of sequences among the global *var* gene population.

The VSA gene families of *P. falciparum* are located in subtelomeric regions and internal clusters. The boundaries between the VSA containing areas and the stable core genome have recently been newly defined by Otto et al. [11], through the analysis of 10 newly cultured field isolates from different geographic regions, by long read Pacific Bioscience sequencing technology. The beginning of the subtelomeric region was defined as the point were newly assembled genomes stop aligning with the 3D7 reference genome, however recombination within the subtelomeric regions was not able to be assessed because the analysed strains were not genetically related. In contrast in this work the analysis of the 3D7-type subtelomeric and central areas of the E5 genome with short and long read WGS enabled an assessment of recombination in the VSA harbouring parts of the E5 genome. Analysis of the 3D7-like subtelomeres and internal clusters by short read WGS exhibited moderate SNP frequency and low coverage and thus suggested relatively frequent sequence alterations compared to the 3D7 refrence sequence. This likely reflects the difficulty of short read sequencing technology in the characterization of DNA sequences with high AT content and an abundance of repetitive DNA elements. In contrast long read WGS data of the subtelomeres and central clusters only identified one large scale recombination event showing that most of the 3D7-type subtelomeric sequences were indeed co-linear with the original 3D7 sequences. Together these data indicate that the majority of subtelomeres of *P. falciparum* are highly conserved across progeny from genetic crosses and that long read sequencing technology is more appropriate for the characterization of the genome areas harbouring VSA gene families.

3D7 and E5 both originate from the same NF54 culture and, therefore, have been in tissue culture for approximately the same time. The highly conserved nature of the E5 genome parts harbouring the 3D7-VSA gene families suggests that mitotic non-chromosomal recombination alone is insufficient to explain the global genetic diversity of the *var* gene family [65]. This suggests that the selective pressure of the host immune system is essential for the expansion of parasite populations with new chimeric *var* genes and thus for the generation of the seemingly endless diversity of the global *var* gene repertoire. Furthermore, the high degree of genetic diversity in the *rifin/stevor* gene families indicates that these non-PfEMP1 VSAs may be under similar diversifying selection as the *var* gene family [29–32].

Larger studies of progeny from natural genetic crosses with long read sequencing technology are necessary to examine the possible role of acquired immunity in the generation the *var* gene and *rifin/stevor* genetic diversity at the population level.

While there has been a long standing interest in the analysis of VSA families from different laboratory strains, recently field isolate VSA gene families have moved into the focus. In this context it has become clear that progeny of natural genetic crosses that show IBD are far more prevalent than previously thought [66].

In order to establish a method that can reliably differentiate between different progeny of a natural genetic cross, a set of 84 MS primers from the NIH database was evaluated for its ability to identify the 3D7 and non-3D7 parts of the E5 genome. 27 MS primers resulted in erroneous genotyping with the PCR conditions applied in this work. This is likely due to the fact that one standardized set of PCR conditions was applied for all primers and no attempts to optimize individual reaction conditions were made. However, even with these standard PCR conditions, 54 of 57 MS genotyping results were confirmed by WGS. 3 MS loci (*ebp, hrp2* and C12M30) showed the same alleles in E5 and 3D7, despite being located in the non-3D7 part of E5. Two of these MS were located within the open reading frames of *ebp* and *hrp2* indicating that these genes are not sufficiently diverse to distinguish between sibling parasites.

Comparative genotyping of E5 and 3D7 with 54 MS genotyping was accomplished within a few days and the use of different fluorophores for different MS on each chromosome enabled “head to head” genotyping of individual E5 and 3D7 chromosomes by multiplex PCR-reactions. This is the first time that MS genotyping has been directly compared to WGS. MS length differences of < 3 bp diffrences between E5 and 3D7 correctly identified the 3D7-type parts of the E5 genome. In some of these 3D7-type MS alleles the PCR fragment length differed from the in silico length of the respective MS in the 3D7 genome (version 3). This is most likely due to DNA slippage during PCR DNA replication. However, given the fact the PCR fragment length of these MS were identical after amplification of E5 and 3D7 this phenomenon appears to be higly reproducible and does not lead to erroneous genotyping.

Recently, Figan et al. [36] identified a set of 12 different microsatellite markers that reliably distinguish between progeny of 4 different experimental genetic crosses. The PCR conditions employed by Figan et al. and the PCR conditions in this work were almost identical suggesting that the two primer sets could be combined for rapid genotyping of field isolates.

SNP barcoding has recently emerged as a genome wide typing technique and has been used to investigate *Plasmodium* and the origin of its genotypes [67, 68]. The barcoding genotyping technique, which is based on a 23 single nucleotide polymorphisms (SNPs) and on high-quality raw sequence data [69], detects differences in the organelle genomes of *P. falciparum* and thus is not suitable for characterization of chromosomal inheritance. Similarly, another SNP assay developed some years earlier, is based on 24 SNP loci that are distributed unevenly across the genome, i.e. some chromosomes do not have SNP markers and others only 1 marker, thus tracking chromosomal cross over events is not possible [70].

SNP and WGS analysis are expensive and depend on the availability of high quality sequence data as well as extensive bioinformatic expertise. Therefore SNP and WGS can only be applied to subsets of *P. falciparum* lines and are usually carried out in specialized centres with extensive resources. In contrast MS genotyping and data analysis can be carried out in smaller centres, potentially enabling investigator driven analysis and identification of *P. falciparum* strains most suitable for subsequent WGS analyses in specialized centres.

The vast majority of the confirmed 54 MS are located in the non-coding parts of the *P. falciparum* genome. Consequently, they are not under purifying selection and may reflect the underlying genetic plasticity of the *P. falciparum* genome more accurately than methods that are based on the detection of SNPs of coding regions.

Future analysis of natural *P. falciparum* cross progeny from semi-immune and non-immune individuals may allow insights into the factors that drive crossover and and non-crossover recombination in *P. falciparum*. In this context MS genotyping may be used to determine IBD in field isolate progeny and to identify parasites clones most suitable for WGS analysis.

## Conclusion

The data presented in this work show that the *var* and *rifin/stevor* gene families represent the most diverse parts of the *P. falciparum* genome, but that the majority of the VSA genes are inherited without alteration in a Mendelian fashion. Furthermore, MS genotyping data correlate well with WGS data suggesting that MS genotyping can be employed to define IBD in progeny of natural *P. falciparum* crosses.

## Additional files

**Additional file 1.** MS Primers that generated PCR products that could not be aligned to the 3D7 MS refrence sequence.

**Additional file 2.** Singelton genes within the E5 genome.

**Additional file 3.** ACT view showing a miss-assembly between E5 and 3D7 in the first *var* gene cluster of chromosome 4. The blue bars at the top represent the E5 bin contig, matching to an area on E5.

**Additional file 4.** ACT screenshot of *var* chimera, box. The top sequence (chromosome 8 of PfE5) is identical to Pf3D7 (middle track, chromosome 8), but does not finish with a telomer. The sequence left hand site of the *var* gene in 3D7 up to the chromosome end (telomer repeat marked with T) is shared to chromosome 14 of PfE5 (lowest track). The black blast hits between the identity of 95–100%. *For visualisation reasons, the chromosome 14 of PfE5 was complemented. So the *var* chimera in PfE5 is on the left hand site of chr14 and on the forward strand.
