# Supplementary figures and images for: Whole genome sequencing and microsatellite analysis of the Plasmodium falciparum E5 NF54 strain show that the var, rifin and stevor gene families follow Mendelian inheritance
Source: Malar J. 2018 Oct 22;17:376. doi: 10.1186/s12936-018-2503-2 (PMC6198375; doi:10.1186/s12936-018-2503-2)

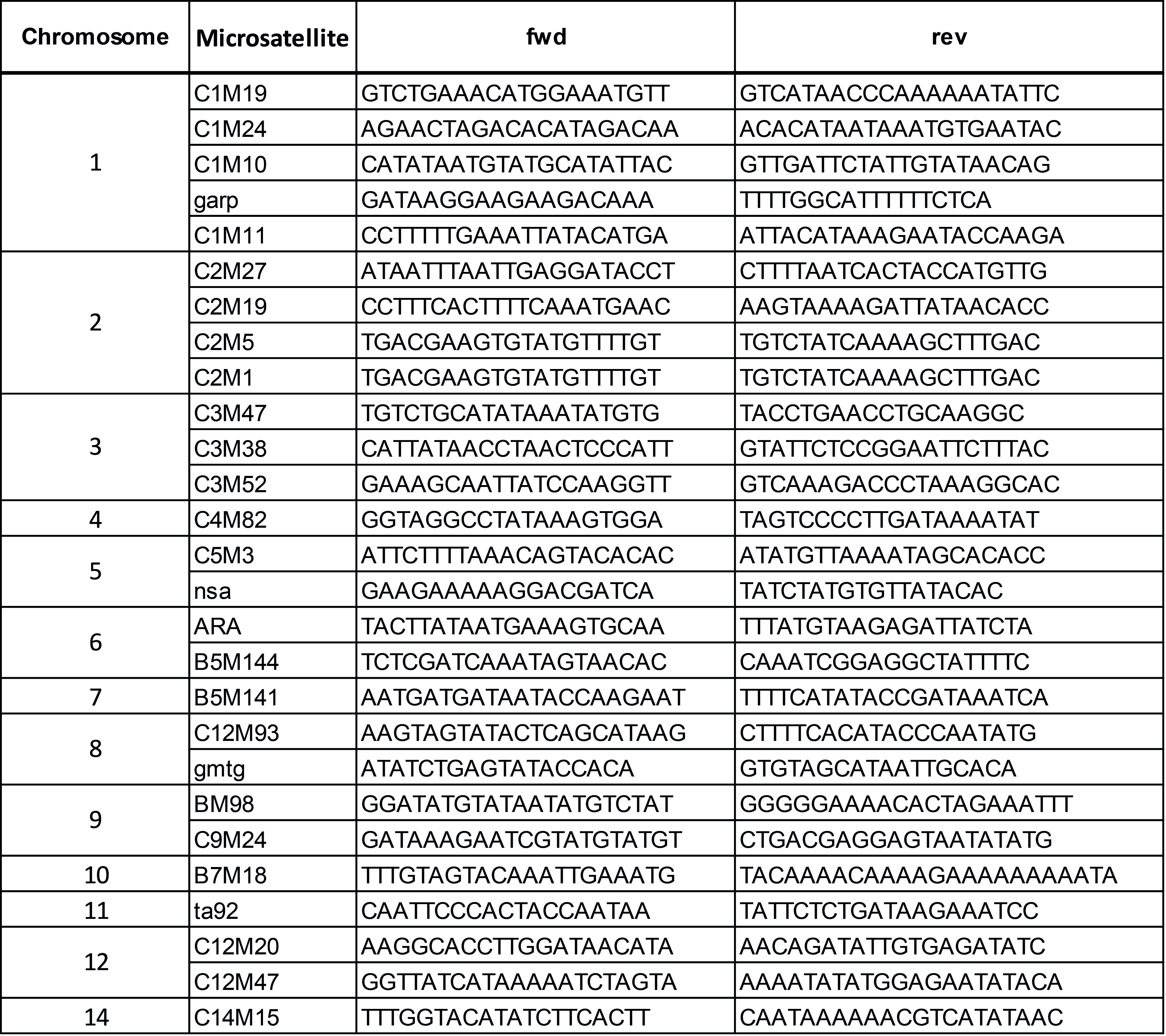

Supplement: Supplementary file 1 — Additional file 1. MS Primers that generated PCR products that could not be aligned to the 3D7 MS refrence sequence. [file 12936_2018_2503_MOESM1_ESM.jpg]

## Slide 1
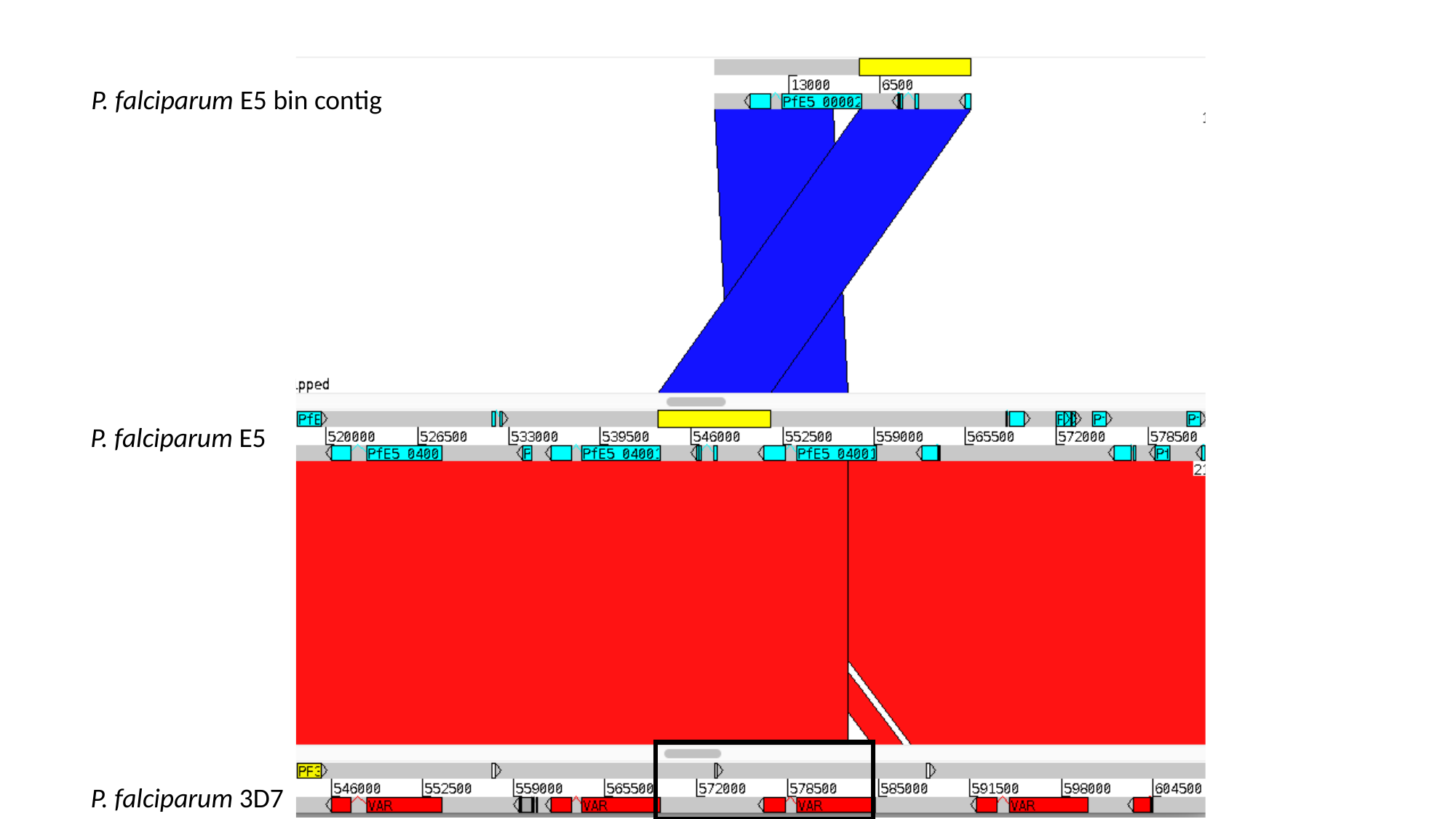

P. falciparum E5 bin contig
P. falciparum E5
P. falciparum 3D7

Supplement: Supplementary file 3 — Additional file 3. ACT view showing a miss-assembly between E5 and 3D7 in the first var gene cluster of chromosome 4. The blue bars at the top represent the E5 bin contig, matching to an area on E5. [file 12936_2018_2503_MOESM3_ESM.pptx]

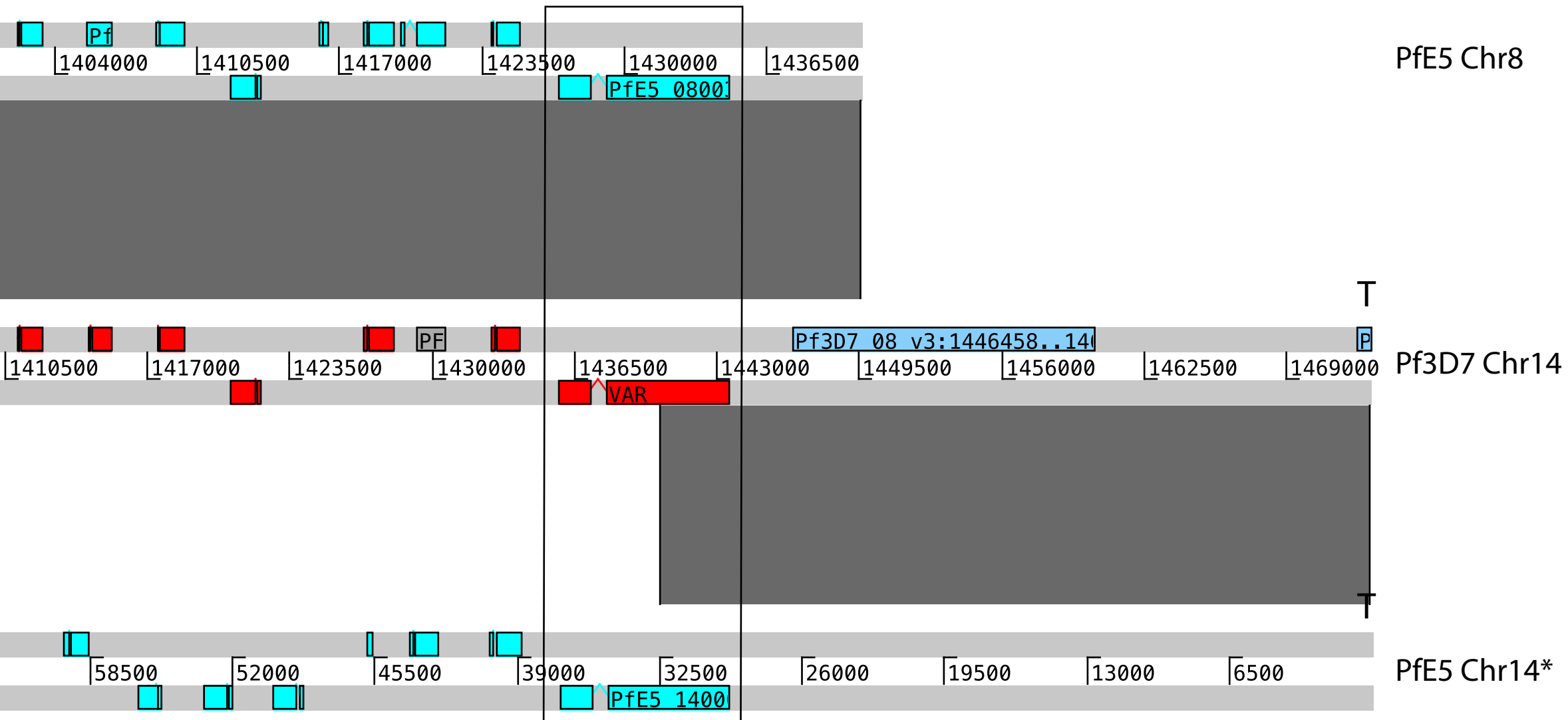

Supplement: Supplementary file 4 — Additional file 4. ACT screenshot of var chimera, box. The top sequence (chromosome 8 of PfE5) is identical to Pf3D7 (middle track, chromosome 8), but does not finish with a telomer. The sequence left hand site of the var gene in 3D7 up to the chromosome end (telomer repeat marked with T) is shared to chromosome 14 of PfE5 (lowest track). The black blast hits between the identity of 95–100%. *For visualisation reasons, the chromosome 14 of PfE5 was complemented. So the var chimera in PfE5 is on the left hand site of chr14 and on the forward strand. [file 12936_2018_2503_MOESM4_ESM.pdf]
