# Supplementary material for: Whole genome sequencing and microsatellite analysis of the Plasmodium falciparum E5 NF54 strain show that the var, rifin and stevor gene families follow Mendelian inheritance
Source: Malar J. 2018 Oct 22;17:376. doi: 10.1186/s12936-018-2503-2 (PMC6198375; doi:10.1186/s12936-018-2503-2)
Supplement: Supplementary file 2 — Additional file 2. Singelton genes within the E5 genome. [file 12936_2018_2503_MOESM2_ESM.pptx]

## Slide 1
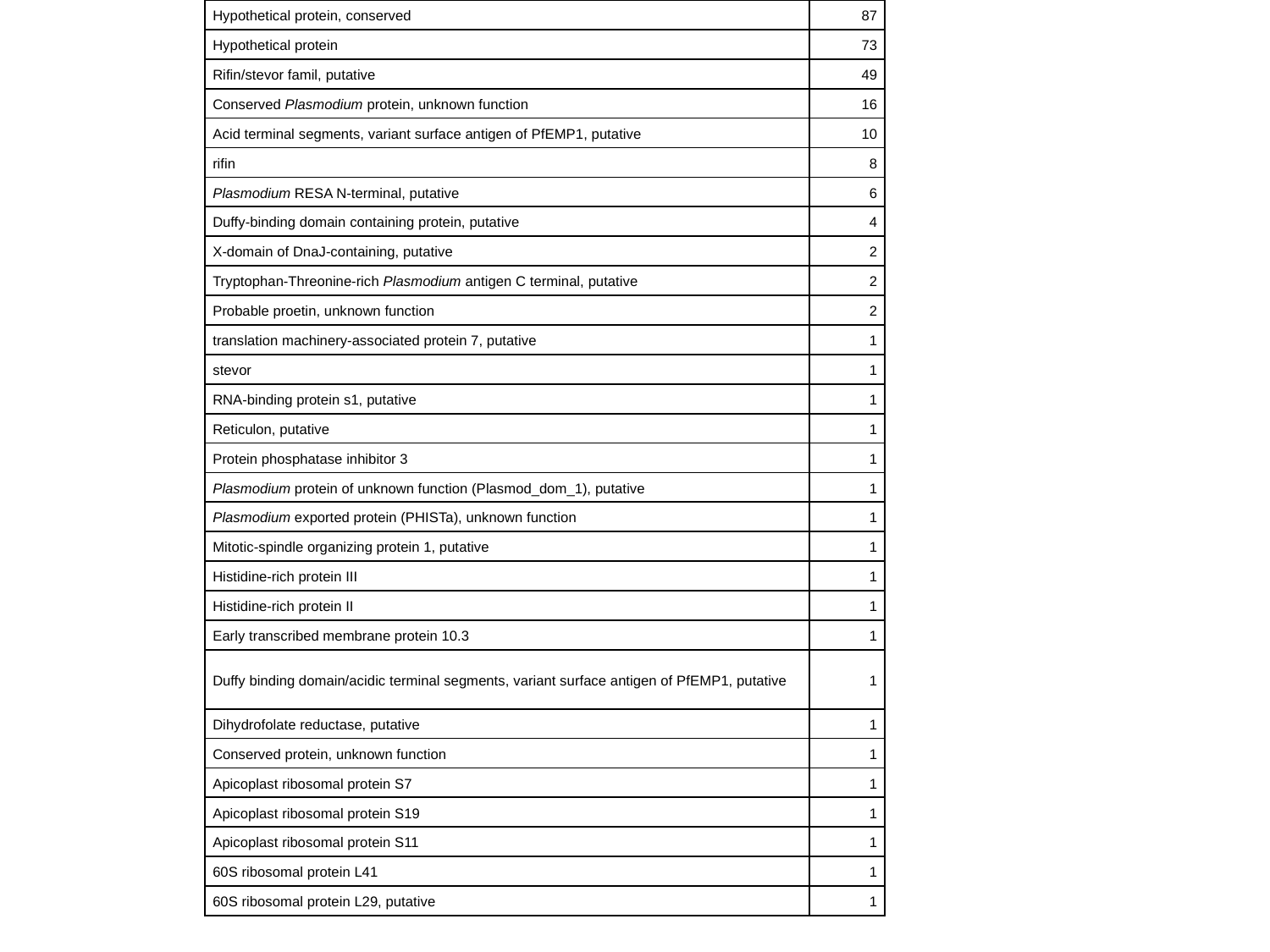

| Hypothetical protein, conserved | 87 |
| --- | --- |
| Hypothetical protein | 73 |
| Rifin/stevor famil, putative | 49 |
| Conserved Plasmodium protein, unknown function | 16 |
| Acid terminal segments, variant surface antigen of PfEMP1, putative | 10 |
| rifin | 8 |
| Plasmodium RESA N-terminal, putative | 6 |
| Duffy-binding domain containing protein, putative | 4 |
| X-domain of DnaJ-containing, putative | 2 |
| Tryptophan-Threonine-rich Plasmodium antigen C terminal, putative | 2 |
| Probable proetin, unknown function | 2 |
| translation machinery-associated protein 7, putative | 1 |
| stevor | 1 |
| RNA-binding protein s1, putative | 1 |
| Reticulon, putative | 1 |
| Protein phosphatase inhibitor 3 | 1 |
| Plasmodium protein of unknown function (Plasmod\_dom\_1), putative | 1 |
| Plasmodium exported protein (PHISTa), unknown function | 1 |
| Mitotic-spindle organizing protein 1, putative | 1 |
| Histidine-rich protein III | 1 |
| Histidine-rich protein II | 1 |
| Early transcribed membrane protein 10.3 | 1 |
| Duffy binding domain/acidic terminal segments, variant surface antigen of PfEMP1, putative | 1 |
| Dihydrofolate reductase, putative | 1 |
| Conserved protein, unknown function | 1 |
| Apicoplast ribosomal protein S7 | 1 |
| Apicoplast ribosomal protein S19 | 1 |
| Apicoplast ribosomal protein S11 | 1 |
| 60S ribosomal protein L41 | 1 |
| 60S ribosomal protein L29, putative | 1 |
